# Potential of Red, Green and Brown Seaweeds as Substrates for Solid State Fermentation to Increase Their Nutritional Value and to Produce Enzymes

**DOI:** 10.3390/foods11233864

**Published:** 2022-11-30

**Authors:** Marta Ferreira, José Manuel Salgado, Helena Fernandes, Helena Peres, Isabel Belo

**Affiliations:** 1 Centre of Biological Engineering, University of Minho, Campus de Gualtar, 4710-057 Braga, Portugal; 2 LABBELS—Associate Laboratory, 4710-057 Braga, Portugal; 3 Biotecnia Group, Department of Chemical Engineering, Campus Agua, University of Vigo (Campus Ourense), As Lagoas s/n, 32004 Ourense, Spain; 4 CIMAR/CIIMAR—Centro Interdisciplinar de Investigação Marinha e Ambiental, Universidade do Porto, Terminal de Cruzeiros do Porto de Leixões, Av. General Norton de Matos, 4450-208 Matosinhos, Portugal; 5 Departamento de Biologia, Faculdade de Ciências, Universidade do Porto, Rua do Campo Alegre, Edifício FC4, 4169-007 Porto, Portugal

**Keywords:** chemical composition, enzyme activities analysis, fatty acids analysis, carbohydrases, filamentous fungi, Fourier Transform Infrared Spectroscopy

## Abstract

Seaweeds are valuable feedstocks with the potential to be used as ingredients in aquafeeds. However, their use are still limited, given their recalcitrant polysaccharide structure. To break this structure, a biotechnological approach such as solid-state fermentation (SSF) by filamentous fungi can be used, which simultaneously increases the nutritional value of the biomass. However, SSF has hardly been studied in seaweeds; thus, in this study, five different seaweeds *(Gracilaria* sp., *Porphyra dioica*, *Codium tomentosum*, *Ulva rigida*, and *Alaria esculenta*) were used as substrates in SSF with *Aspergillus ibericus* MUM 03.49 and *A. niger* CECT 2915. Firstly, the seaweeds were fully characterized, and, then, changes in the crude protein and carbohydrate contents were assessed in the fermented biomass, as well as any carbohydrases production. The SSF of *U. rigida* with both fungi resulted in the maximum xylanase and β-glucosidase activities. The maximum cellulase activity was achieved using *Gracilaria* sp. and *U. rigida* in the SSF with *A. niger*. The protein content increased in *C. tomentosum* after SSF with *A. ibericus* and in *U. rigida* after SSF with both fungi. Moreover, *U. rigida’s* carbohydrate content decreased by 54% and 62% after SSF with *A. ibericus* and *A. niger*, respectively. Seaweed bioprocessing using SSF is a sustainable and cost-effective strategy that simultaneously produces high-value enzymes and nutritionally enhanced seaweeds to be included in aquafeeds.

## 1. Introduction

Seaweeds, also called macroalgae, are a group of autotrophic plant-like organisms without leaves, roots, and stems [1]. In 2020, 36 million tons (wet weight) of seaweeds were produced, of which 97 percent originated from aquaculture, which was worth USD 16.5 billion [2].

The high biomass yield, fast growth rate, positive carbon balance, and lack of competition for freshwater and arable land with terrestrial plants are significant advantages that support the sustainable and economical use of seaweeds [3].

There are three large groups of seaweeds based on their photosynthetic pigmentation: green (*Chlorophyta*), red (*Rhodophyta*), and brown (*Phaeophyta*) [4]. Seaweeds contain 80–90% water, 50% (dry weight, DW) carbohydrates, 1–3% DW lipids, and 7–38% DW minerals [5]. The protein content varies greatly among groups, from 10 to 47% DW, constituted by a high amount of essential amino acids [5]. Specifically, the main photosynthetic pigments of green seaweeds are chlorophylls a and b, xanthophyll and carotene [6], and the species from the *Ulva* genus are constituted by ulvan, a sulfated polysaccharide present in their cell walls [7]. Chlorophylls a and d and β-carotene are pigments present in red seaweeds, whose color is due to the presence of phycoerythrin and phycocyanin [6]. The major cell-wall polysaccharides of red seaweeds are galactans, such as carrageenan and agar [8], while in brown seaweeds, they are fucoidan, laminarin, alginate, and mannitol [9,10]. The chlorophylls a and c, fucoxanthin, and β-carotene are the pigments found in brown seaweeds [6]. Nonetheless, a wide range of other bioactive compounds can also be found in all types of seaweeds, such as phenolic and antioxidant compounds, vitamins (A and B complex), minerals (calcium, potassium, magnesium, sodium, and others), and polyunsaturated fatty acids [11]. 

Seaweeds are already used in different applications worldwide, such as for industrial phycocolloids extraction, human and animal nutrition (livestock), or farming (biofertilizers) [12]. Despite the seaweeds’ promising nutritional composition and presence of bioactive substances, their nutritional value is still underexplored, mainly because of their structural polysaccharides, which may be overcome through valorization strategies, such as solid-state fermentation (SSF).

SSF is a bioprocess that occurs in a solid substrate in the absence or near-absence of free water. However, the substrate must contain sufficient moisture to allow microbial growth and metabolism [13]. SSF may be applied in a wide range of raw materials, from agro-industrial wastes to seaweeds, for example. In fact, seaweeds may be used as both physical and as nutritional substrates resulting, at the end of SSF, in a nutritionally enhanced biomass and/or a functional extract with bioactive properties. Furthermore, seaweeds may be used in SSF without drying, since their moisture content (around 70–90%) is suitable for fungi growth. During the SSF of seaweeds, filamentous fungi may produce cellulase [14], fucoidanase, alginate lyase [15], pectinase [16], and xylanolytic and lignocellulolytic enzymes [17,18], hydrolyzing their structural polysaccharides and releasing oligosaccharides and free sugars [14]. Moreover, SSF may increase seaweed’s protein content [19,20,21]. 

Therefore, SSF may be successfully applied in seaweeds, improving their digestibility and nutritional value, simultaneously reducing the structural polysaccharide matrix and increasing the protein content. Moreover, fungal biomass and exogenous enzymes also enrich the fermented biomass, as proven by the few works previously reported [17]. The dietary inclusion of seaweeds in fish feeds is still limited, due to their high non-starch polysaccharide (NSP) content, such as cellulose, xylans, and phycocolloids (agar, carrageenan, and alginate), which are anti-nutritional factors that negatively impact the digestion of monogastric animals, including fish [22]. These animals do not have non-starch polysaccharide-degrading enzymes, such as β-glucanases or β-xylanases, that digest NSPs [22,23]. Indeed, high dietary NSP levels increase the digesta viscosity, altering its transit velocity rate, gut microbiota, and intestinal morphology, decreasing intestinal nutrient absorption [24]. The dietary inclusion of up to 10% of seaweeds in aquafeeds showed promising results on growth [25,26] and fish immune status [27]. However, incorporation levels above 10% reflected negative effects on fish growth, and feed and protein utilization [28,29]. The utilization of SSF in *U. rigida*, a green seaweed, within a circular economy approach towards its inclusion in aquafeeds, was recently studied [17]. The dietary inclusion of 5% of fermented *U. rigida* improved the feed efficiency without affecting the growth of European seabass (*Dicentrarchus labrax*), while the dietary inclusion of crude seaweed decreased the fish growth and feed efficiency [30]. Thus, the SSF of seaweeds may effectively result in enhanced nutritional value ingredients and/or be used to obtain value-added additives for fish feeds.

Therefore, this study aims to characterize the chemical composition of different red, green, and brown seaweeds, including their polysaccharide content, fatty acid profile, and Fourier Transform Infrared Spectroscopy (FTIR) analysis, and to assess their potential as substrates in SSF without any previous treatment or nutrient supplementation during the SSF. Furthermore, the SSF was studied using two filamentous fungi, evaluating the NSP-degrading enzyme production, the protein content, and the structural alteration in the polysaccharide matrix of seaweeds at the end of the SSF.

## 2. Materials and Methods

### 2.1. Seaweeds and Microorganisms

The red algae *Gracilaria gracilis* and *Porphyra dioica*, green algae *Codium tomentosum* and *Ulva rigida*, and brown alga *Alaria esculenta* were provided whole and dried by *Algaplus*, an IMTA-producer seaweeds company (Aveiro, Portugal). The seaweeds were milled, stored in hermetic plastic bags, and kept in the dark at room temperature until use. 

The *Aspergillus ibericus* MUM 03.49 was obtained from Micoteca, of the University of Minho (Braga, Portugal), and the *Aspergillus niger* CECT 2915 was obtained from Colección Española de Cultivos Tipo (CECT, Valencia, Spain). Both fungi were preserved in glycerol stocks at −80 °C. Then, the strains were revived on potato dextrose agar (PDA) at 25 °C for 6 days. 

### 2.2. Seaweeds Chemical Characterization 

The chemical composition of the seaweeds was assessed before and after the SSF, following the Association of Official Analytical Chemists’ (AOAC) methods [31]: the moisture by drying samples in an oven at 105 °C for 24 h (AOAC, 934.01); the ash by incineration at 550 °C for 2 h in a muffle furnace (AOAC, 930.05); the total protein content by the Kjeldahl method (AOAC 981.10) with a Kjeltec system (Foss 8400), using the N content and the protein factor (k_p_) of 5 [32]; the lipid content by mixing 0.4 g of the dry sample with 6 mL of chloroform/methanol (2:1; *v*/*v*), for 1 h, recovering the lower phase (chloroform with lipids), and evaporating the chloroform using a nitrogen stream; the salts content by washing the seaweeds with distilled water for 24 h, followed by evaporation at 60 °C for 24 h and incineration in a muffle furnace at 550 °C for 2 h; the total carbohydrates by quantitative acid hydrolysis (QAH) in a two-step acid treatment. Briefly, the samples were digested with 72% (*w*/*w*) H_2_SO_4_ (30 °C for 1 h), and then diluted to 4% (*w*/*w*) with distilled water and placed at 121 °C for 1 h. After cooling to room temperature, the samples were filtered through a Gooch crucible to retain the insoluble phase, reported as the acid-insoluble residue (AIR), and placed in a hot air oven at 105 °C. The filtrate was analyzed by a high-performance liquid chromatography (HPLC) system with a Jasco830-IR intelligent refractive-index detector and a Varian MetaCarb 87H column. The column was eluted with 0.005 M H_2_SO_4_, and the flux was 0.6 mL/min at 60 °C. 

An extraction with distilled water using a solid:liquid ratio of 1g:5 mL was performed to estimate the free reducing sugars, soluble protein, and total phenolic content. Briefly, the extraction was performed with constant mechanical agitation for 30 min at room temperature. The mixture was filtered using a nylon net, and the liquid fraction was centrifuged (7000 rpm, 10 min, 4 °C) and stored at −20 °C until the analysis. The soluble protein was determined by the Bradford method [33]. The free-reducing sugars were determined using the 3,5-dinitrosalicylic acid method (DNS) [34]. The total phenols content (TPC) was measured by the Folin–Ciocalteau method [Commission Regulation (EEC) No. 2676/90], and TPC units were defined as g of gallic acid equivalents (GAE) per kg of dry seaweed. The total organic carbon was measured using a Thermo Finnigan Flash Element Analyzer 1112 series (San Jose, CA, USA), and the minerals were determined in the ashes using Flame Atomic Absorption and Atomic Emission Spectrometry (FFAS/FAES; FAAS/FAES).

### 2.3. Fatty Acids Analysis

The fatty acid methyl esters (FAMES) production was carried out using the method proposed by Ferreira et al [35]. The analysis of FAMES was carried out in a gas chromatography (GC) system (VARIAN 3800) equipped with a flame ionization detector (FID). The FAMEs were separated using a TRB-WAX 30 m × 0.25 mm × 0.25 μm column (TR140232, Teknokroma, Tr-wax, Sant Cugat del Vallès, Spain) with helium as the carrier gas at 1.0 mL/min. The airflow was settled at 250 mL/min, and the nitrogen and hydrogen flow at 30 mL/min. The injection port and detector temperatures were 250 and 280 °C, respectively. The initial oven temperature was 40 °C for 2 min, with a 30 °C/min ramp to 150 °C and a 3 °C/min final ramp to 250 °C. Calibration curves were carried out for each fatty acid, and the results were expressed as mg/g of dry seaweed.

### 2.4. Fourier Transform Infrared Spectroscopy (FTIR)

The samples of the milled and dried seaweeds were analyzed using a Fourier Transform Infrared Spectroscopy (FTIR). An ALPHA II- Bruker spectrometer (Ettlingen, Germany), with a diamond-composite attenuated total reflectance (ATR) cell was utilized. The FTIR spectra were recorded between 400 and 4000 cm^−1^; all the spectra resulted from the average of three measurements, with 64 scan cycles per sample with 4 cm^−1^ resolution.

### 2.5. Solid-State Fermentation (SSF) of Seaweeds

The SSF was carried out in 500 mL Erlenmeyer flasks with 5 g of each dried seaweed. The moisture was adjusted to 75% (*w*/*w*, wet basis) with distilled water and sterilized (121 °C, 15 min). The fungi were suspended in a sterile solution (1 g/L peptone and 0.1 g L^−1^ Tween 80), and the spores’ concentration was adjusted to 10^6^ spores/mL. Each seaweed was inoculated with 2 mL of the spores’ suspension and incubated at 25 °C for 7 days. At the end of the SSF, the fermented seaweeds were characterized, and the enzymes were extracted with distilled water using a solid:liquid ratio of 1 g:5 mL. The crude extract was stored at −20 °C until analysis.

### 2.6. Enzyme Activities Analysis

The xylanase activity was determined using xylan from beechwood (1%) in a sodium citrate buffer as the substrate (0.05 N, pH 4.8). The samples were incubated at 50 °C for 15 min, and the free sugars were determined by the DNS method. The cellulase activity was similarly determined as described for the xylanase activity, but the substrate used was carboxymethyl cellulose (CMC, 2%), and the samples were incubated at 50 °C for 30 min. For both assays, one unit of xylanase or cellulase activity was defined as the amount of enzyme necessary to release 1 μmol of xylose or glucose per minute, respectively, under the reaction conditions.

The β-glucosidase activity was assessed using 4 mM 4-nitrophenyl b-D-glucopyranoside (pNPG) as the substrate. The samples and substrate were incubated at 50 °C for 15 min. After cooling, sodium carbonate (1 M) and distilled water were added, and the absorbance was read at 400 nm. One unit of β-glucosidase activity was defined as the quantity of enzyme required to release 1 μmol of p-nitrophenol per minute under the reaction conditions.

### 2.7. Statistical Analysis

The results were analyzed by one-way analysis of variance (ANOVA) and, if significant differences were detected (*p* < 0.05), Tukey’s multiple range test was applied to discriminate the means. The statistical analyses and principal components analysis were performed using Statgraphics Centurion XVI.II software (Statgraphics Technologies Inc., The Plains, VA, USA).

## 3. Results

### 3.1. Seaweeds Chemical Composition 

The moisture content of the different seaweeds ranged between 8.54 ± 0.36 (% *w*/*w*) in the dried *Gracilaria* sp. and 15.5 ± 0.13 (% *w*/*w*) in the dried *U. rigida* (Table 1). Drying the seaweeds before bioprocessing is an important step before their application in different industries since they are easily prone to deterioration. Indeed, the reduction of water activity retards microbial growth, helping preserve the seaweeds’ nutritional and functional quality and reducing the storage volume [36]. 

The ash content varied among the five seaweeds from 19.2 ± 0.01 (% *w*/*w*) in the *P. dioica* to 45.5 ± 0.05 (% *w*/*w*) in the *C. tomentosum*. All the seaweeds analyzed in this study showed a lower ash content compared to those reported in the literature [37,38], except for the *A. esculenta*, whose 31.1% (*w*/*w*) ash content was higher than that reported by Mæhre et al. [39] (24.56%). The higher ash content observed in the *C. tomentosum* was probably related to the higher salt content of this seaweed. 

The crude protein content was assessed by converting the total nitrogen to protein using the conversion factor of 5. The traditional conversion factor of 6.25, which is used as the standard factor for several materials, including seaweeds, assumes that protein is the only source of nitrogen in the analyzed sample and, in fact, any plant material, such as macroalgae, has substantial sources of non-protein nitrogen, as chlorophyll, free amino acids, nucleic acids, and inorganic nitrogen [32], which may lead to an overestimation of the protein content. Therefore, in this work, a conversion factor of 5 was considered [32]. The brown seaweed *A. esculenta* had the lowest protein content (12.6 ± 0.16% *w/w*) among all the seaweeds, followed by the green seaweeds *C. tomentosum* and *U. rigida*, which had similar amounts of crude protein (14.2 ± 0.04 and 14.6 ± 0.35% *w*/*w*, respectively). The red seaweeds had higher protein contents than the other seaweeds, especially the *Gracilaria* sp. The crude protein varies between seaweed species, and brown macroalgae usually have lower protein contents than green and red macroalgae [40]. Similar values were reported by Fernandes et al. [17] for U. rigida (16.9% *w*/*w*), by Machado et al. [41] for *P. dioica* (23.7%), by Echave et al. [42] for C. tomentosum (15%), by Neto et al. [38] for *Gracilaria* sp. (23.6%), and by Lytou et al. [43] for *A. esculenta* (11.3% for seaweeds collected in 2019 and 9.1% for seaweeds collected in 2020). 

The carbohydrate content of the five seaweeds ranged between 9.59% (*w*/*w*) in the *Gracilaria* sp. and 33.3% (*w*/*w*) in the *C. tomentosum*. This is quite a variable factor, even within the same algae genus. In the literature, values from 4.71% [44] to 53.54% [45] are reported for the *Gracilaria* species. In general, the carbohydrate content obtained in this study was lower than those reported in other studies [46,47,48]. In this work, the seaweeds were characterized and directly used in the SSF without a previous washing stage. This step could lead to a concentration of other compounds, such as carbohydrates.

The lipid fraction ranged between 1.91 and 6.39% *w*/*w* (Table 1). The green macroalgae *C. tomentosum* presented the highest lipid content among the seaweeds. The same result was reported by Rodrigues et al. [49], who observed that *C. tomentosum* had the highest lipid content (3.6%) among six studied seaweeds. Regarding the lipid content of *Gracilaria* sp., *P. dioica*, and *A. esculenta*, they were higher than those described in other studies, such as 0.7% [38] and 0.3% [50] for *Gracilaria* sp.; 0.86% [51] and 1.2% [52] for *P. dioica*; and 1.1% [53] and 0.89% [54] for *A. esculenta*. In the case of *U. rigida*, its lipid content was similar to that observed by Fernandes et al. [17] (1.4%) and Kumari et al. [55] (2.0%). 

Carbon (C), nitrogen (N), and the C/N ratio are essential factors for SSF since the C supplies the energy necessary for the microorganisms’ growth and metabolism, while the N stimulates fungal conidiation [56]. In this study, the carbon and nitrogen contents of the seaweeds ranged between 21.7–38.1% and 3.03–5.27%, respectively, while the C/N ratios varied between 5.86 (*Gracilaria* sp.) and 12.2 (*A. esculenta*) (Table 1). Karray et al. [57] carried out SSF with *A. niger* CTM 10099 in a mixture of *U. rigida* and wheat bran; they tested β-glucosidase production under several C/N ratios (from 1 to 80) and observed that the highest enzyme activity was obtained using a C/N of 5, decreasing the enzymes’ activity at higher ratio values. 

The micro- and macro-mineral composition of the five seaweeds are presented in Table 2. The most abundant macro-elements found were Ca, Mg, K, and Na, supporting the evidence that these seaweeds are an ideal source of these nutritionally important minerals. Regarding the micro-elements, Zn, Mn, Fe, and Cu are some of the most important, but they were not present in all the macroalgae studied. Fe was present in all the seaweeds and was the most abundant among the micro-elements, except in *A. esculenta,* where Zn had the highest concentration. The green seaweed *U. rigida* was constituted by the highest amounts of Fe, Mn, and Mg among all the seaweeds, values that are even greater than those reported by Cassani et al. [37]. Although macroalgae are known as good sources of selenium, this element was not detected in the seaweeds we studied, which is probably due to the small quantity present. Mæhre et al. [39] observed that selenium levels ranged from 0.02 to 0.53 mg kg^−1^ DW in different macroalgae, where the red algae *Palmaria palmata* and *Vertebrata lanosa* presented the highest amounts.

Despite seaweeds’ low lipid content, their fatty acid profile contains nutritionally important polyunsaturated fatty acids (PUFAs) [55]. The fatty acid composition of the different seaweeds is shown in Table 3. In general, all the seaweeds had a high content of unsaturated fatty acids (54.8%), of which 38.1% were PUFAs and 16.7% were monounsaturated fatty acids (MUFA). On the other hand, the seaweeds’ saturated fatty acids content was around 45.2%. Palmitic acid represents more than half of the total SFA in all seaweeds, ranging between 1.34 mg g^−1^ in *P. dioica* and 14.3 mg g^−1^ in *C. tomentosum*. Indeed, *C. tomentosum* had the highest total fatty acid content (47.8 ± 3.09 mg g^−1^) among all the seaweeds, including PUFAs such as linoleic (5.93 mg g^−1^), alpha-linolenic (ALA; 5.92 mg g^−1^), and docosahexaenoic acids (DHA; 5.66 mg g^−1^). It is also worth noting that DHA was found in all the macroalgae studied. Along with arachidonic acid (ARA) and eicosapentaenoic acid (EPA), DHA is an essential fatty acid for fish nutrition, especially in marine species, since it benefits fish physiological and cardiovascular health, inflammatory and immune responses, and neural development [58,59]. Rodrigues et al. [49] also observed that, among six seaweeds studied, *C. tomentosum* had the highest fatty acid content (27.6 ± 0.15 µg FA/mg dry seaweed), and PUFAs were present in a higher quantity than SFA, as was observed in the present study. 

### 3.2. Production of Enzymes during SSF

In this work, the seaweeds were used as substrates in SSF with two fungi species. Previous results demonstrated that *A. ibericus* MUM 03.49 has a high potential to grow on *U. rigida* under SSF conditions [17], and *A. niger* CECT 2915’s suitability to produce carbohydrases on the SSF of winery, olive mill, and brewery wastes [60]. Table 4 shows the xylanase, cellulase, and β-glucosidase activity obtained from the SSF. The maximum xylanase and β-glucosidase activities were obtained in the SSF of *U. rigida* with both fungi. Nevertheless, for the SSF of *U. rigida*, the higher xylanase activity was achieved with *A. niger*, increasing 5.3-fold this enzyme production compared to the SSF using *A. ibericus*. The cellulase activity was higher in the SSF of *Gracilaria* sp. and *U. rigida* with *A. niger* than in the other seaweeds (*p* < 0.05). The use of seaweeds as a substrate for enzyme production during SSF is still underexplored. Fernandes et al. [17] used *U. rigida* in SSF with *A. ibericus* and obtained 359.8 U g^−1^ of xylanase activity, a higher value than that obtained in this study using the same strain. These authors used micronized seaweed, which may have contributed to higher enzyme activity. This may indicate that the reduced particle size of the substrates is an important parameter for enzyme production during SSF. In addition, reducing sugars, salts, and ashes were present in higher amounts in the dried micronized *U. rigida*, which could help fungal growth and, consequently, enzyme production. Trivedi et al. [14] used the green seaweed *Ulva fasciata* supplemented with minerals as a substrate for cellulase production during SSF with *Cladosporium sphaerospermum*, obtaining 10.2 ± 0.40 U g^−1^ of enzyme. In this study, using *U. rigida* as the substrate, the cellulase activity reached 19.8 ± 1.61 U g^−1^ with *A. ibericus* and 101 ± 10.27 U g^−1^ with *A. niger*. Other works demonstrated that other enzymes may also be produced during semi-solid state fermentation (SSF) of seaweeds, such as agarase [61] and pectinase [16]. The mixture of seaweeds with wastes has also been studied for enzyme production by SSF: Karray et al. [57] mixed *U. rigida* with wheat bran to produce β-glucosidase, and Mohapatra [62] used *Sargassum* waste along with cow dung to produce alginate lyase and mannanase. The production of rhamnase in green seaweeds, agarase in red seaweeds, and alginate lyase in brown seaweed were also assessed in this study, but no enzymatic activities were detected.

### 3.3. Variation of Seaweed Nutritional Composition after SSF

**Figure 1:**
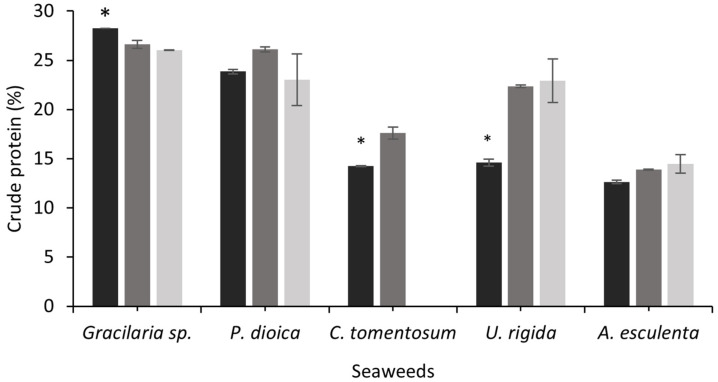
Crude protein content of unfermented seaweeds (black bars) and fermented seaweeds with *A. ibericus* MUM 03.49 (gray bars) and *A. niger* CECT 2915 (light gray bars). Results are presented as the mean (n = 3) ± SD. * denotes significant differences between unfermented and fermented seaweeds (Tukey’s test; *p* < 0.05) comparing each seaweed species.

The nutritional quality of fermented seaweeds was evaluated in the present study. Figure 1 shows the seaweed protein content before and after SSF with both fungi. The protein content of *C. tomentosum* and *U. rigida* increased after SSF with *A. ibericus* (*C. tomentosum*) and both fungi (*U rigida*; *p* < 0.05). Fernandes et al. [17] observed an increase of 1.3-fold in the protein content of *U. rigida* after SSF with *A. ibericus*. In this study, the *U. rigida* protein increased 1.5-fold and 1.6-fold with *A. ibericus* and *A. niger*, respectively. Other authors used SSF in different seaweeds, also aiming to increase the protein content: Loaiza-Bonilla et al. [21] performed a hydrothermal pretreatment followed by SSF of *Sargassum* with *Aspergillus oryzae*, achieving a higher protein content after 96 h (6.6%) compared to the control (3.7%); the SSF of *Sargassum fulvellum* mixed with palm kernel cake increased the protein content from 3.75% to 5.23% [19]. In this study, the *P. dioica* and *A. esculenta* protein contents did not vary after fermentation. On the other hand, a decrease in the crude protein during the SSF with both fungi was observed for *Gracilaria* sp. Sousa et al. [56] also observed a protein reduction after SSF of sunflower, rapeseed, and soybean cakes with *Rhyzopus oryzae* and *A. ibericus*, concluding that these fungi may use the available N to support their growth, since no N supplementation was carried out.

**Figure 2:**
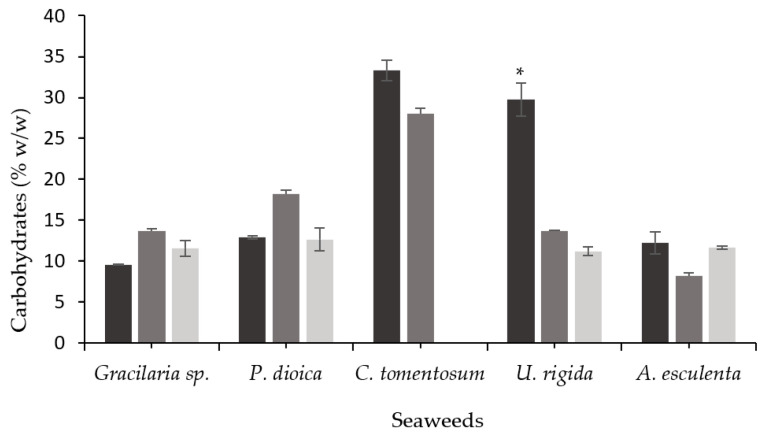
Carbohydrate content of unfermented seaweeds (black bars) and fermented seaweeds with *A. ibericus* MUM 03.49 (gray bars) and *A. niger* CECT 2915 (light gray bars). Results are presented as the mean (n = 3) ± SD. * denotes significant differences between unfermented and fermented solids (Tukey’s test; *p* < 0.05) comparing each seaweed species.

During SSF, the filamentous fungi partially degrade the substrate’s structural matrix through the production of carbohydrate-degrading enzymes [63]. The carbohydrate content before and after the seaweeds’ SSF is shown in Figure 2. The *U. rigida* carbohydrate content decreased by 54% (*A. ibericus*) and 62% (*A. niger*) after SSF (*p* < 0.05), while no differences were observed for the other seaweeds before and after SSF. As previously observed, the SSF of *U. rigida* also resulted in the highest enzyme production among all the seaweeds. According to Fernandes et al. [17], the SSF of unwashed *U. rigida* with *A. ibericus* led to a higher total polysaccharide reduction, along with higher cellulase and xylanase production. The same correlation between polysaccharide reduction and enzyme production was also observed using other substrates in SSF, such as brewer’s spent grain [63], or soybean and rapeseed cakes [56].

### 3.4. FTIR-ATR Characterization of Unfermented and Fermented Seaweeds

**Figure 3:**
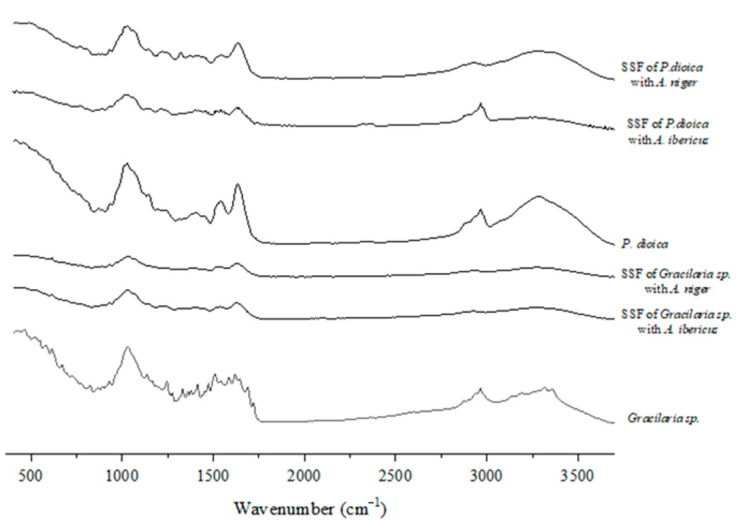
FTIR-ATR spectra of red seaweeds.

**Figure 4:**
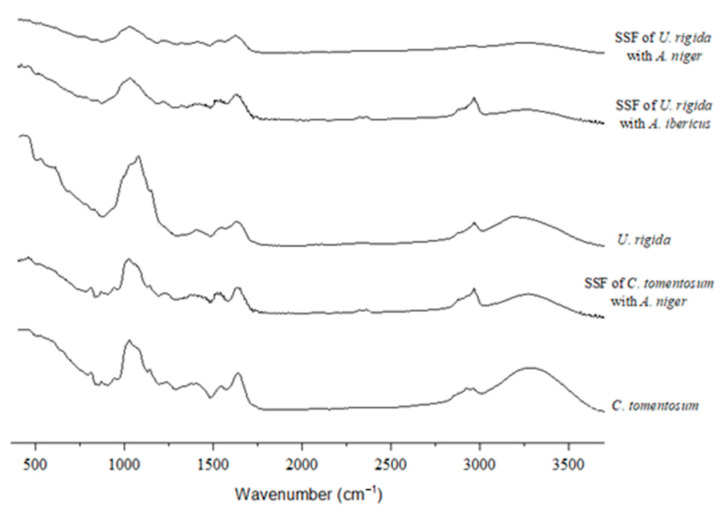
FTIR-ATR spectra of green seaweeds.

**Figure 5:**
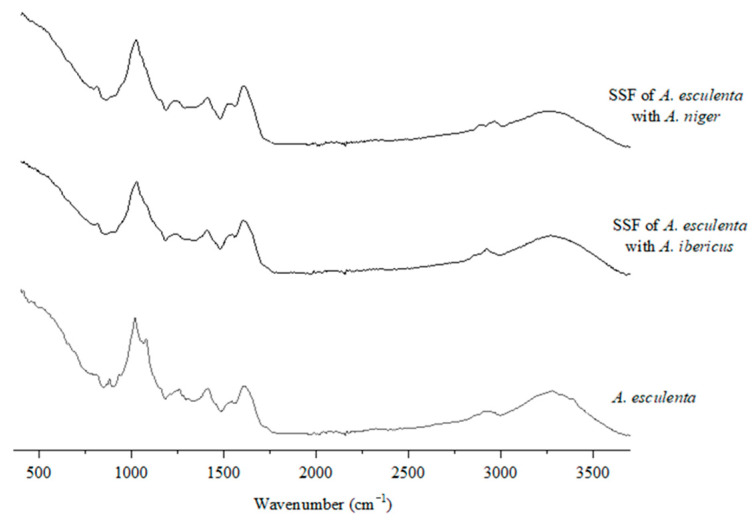
FTIR-ATR spectra of brown seaweed.

The FTIR spectra (Figure 3, Figure 4 and Figure 5) were studied to assess the structural changes in the seaweed biomass after the SSF. In general, all the unfermented samples showed similar characteristics to polysaccharide- and protein-rich materials, including a broad band of 3500–3200 cm^−1^, associated with O–H and N–H stretching, bands of 3000–2900 cm^−1^, assigned to C–H stretching, and also several minor bands of 1300–1100 cm^−1^, associated with the C-O-C stretching vibrations of polysaccharides [64]. Overall, these bands intensity decreased in the fermented samples, which indicates that the fungal growth and action upon the substrate may modify their polysaccharide structure.

Typical bands of agar samples were identified in the unfermented *Gracilaria* sp. (Figure 3): the 930 cm^−1^ band is related to the C–O vibration of LA units, the 1250 cm^−1^ band is assigned to the S=O stretching vibration, and the 1370 cm^−1^ band, which is related to the ester sulfates [65]. In the fingerprint region (900–750 cm^−1^ band), there is a band of 890 cm^−1^ associated with the C–H present in the anomeric carbon of the β-galactose (unsulphated β-D-galactose) bonds (also characteristic of agar samples), and a band of 824 cm^−1^ associated with the presence of sulfated galactose units in the C-2 position, which can also be observed in an agaran-type polysaccharide isolated from the red seaweed *Grateloupia filicina* [66], and in the lambda carrageenan standard spectra [67]. The intensity of the bands decreased in the spectra from the fermented *Gracilaria* sp. with both fungi.

The band from 3000 cm^–1^ to 2800 cm^−1^ in the FTIR spectra of *P. dioica* is similar in this seaweed before and after the SSF with *A. ibericus*, indicating that the SSF with *A. niger* induced deeper modifications in this seaweed’s structure (Figure 3). The band at 1624 cm^–1^ is linked to the O–H stretching of bonded water [68], and there are also two less intense peaks at 929 cm^−1^ and 867 cm^−1^, which correspond to the C–O vibrations of the 3,6-anhydro-D-galactose bonds and the C–O–SO_3_ on the C-6 bonds of galactose, respectively [69]. 

The unfermented and fermented *C. tomentosum* spectra are very similar (Figure 4), indicating that the SSF may not have affected the structural composition of this seaweed. Indeed, the SSF of *C. tomentosum* resulted in the lowest enzymatic activities among all the seaweeds, which can probably be explained by the lower development of the fungus on this substrate. Both the bands at 1640 and 1540 cm^–1^ are assigned to the carbonyl stretching of amide, while the 1379 and 1245 cm^–1^ bands correspond to the −CH2–, −CH3, and sulfur groups [70]. The peaks observed at 1143, 1078, and 1054 cm^–1^ may be associated with (1→4)-b-D-mannans links [49]. 

As seen for *P. dioica*, the *U. rigida* FTIR spectra also exhibited a band between 3000 cm^–1^ and 2800 cm^−1^ for both the unfermented and fermented (with *A. ibericus*) samples (Figure 4). The SSF of *U. rigida* with *A. niger* led to higher xylanase and cellulase activities, which probably resulted in a stronger substrate modification. The band found at 1637 cm^−1^ is associated with the vibration of the asymmetric stretching of the carboxyl groups and the presence of uronic acids [71]. The N–H stretching observed at 1544 cm^−1^ is linked to the presence of amino acids, and both bands found between 840 and 790 cm^−1^ are associated with a C=C stretch [72].

The spectra of unfermented and fermented *A. esculenta* are also very similar, indicating that, similarly to what was observed for *C. tomentosum*, both fungi found it more difficult to grow in these substrates (Figure 5). The peak observed at 1620 cm^−1^ in both unfermented and fermented *A. esculenta* could be attributed to the stretching vibration of the COO- present in alginate [69], and the band around 1540 cm^−1^ may be associated with the presence of amide II, confirming the presence of proteins in the samples [64]. The peak found around 1414 cm^−1^ in *A. esculenta* may be associated with the vibration of a C–OH deformation with the contribution of the O–C–O symmetric stretching vibration of the carboxylate group. The broader band observed between 1230 and 1260 cm^−1^ is assigned to the presence of sulphate ester groups (S=O), indicating that *A. esculenta* has fucoidan and other sulphated polysaccharides in addition to alginate in its composition [69]. The presence of guluronic and mannuronic acids can be evidenced by the presence of bands at 1080 and 787 cm^−1^ and at 1019 cm^−1^ and 808 cm^−1^, respectively. These are alginate characteristic bands, and, since the peak at 808 cm^−1^ disappears in the fermented *A. esculenta*, it may indicate that both fungi hydrolyzed this polysaccharide.

### 3.5. Principal Components Analysis

**Figure 6:**
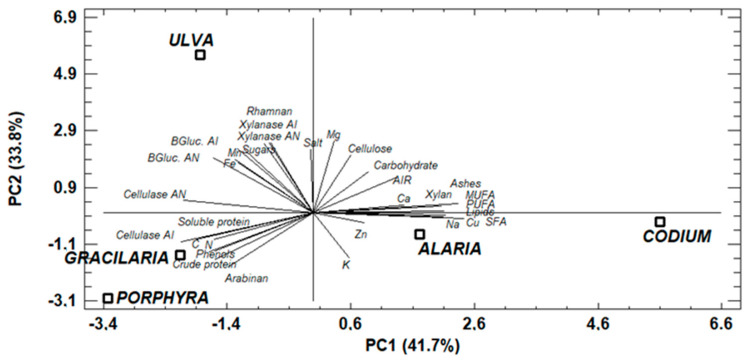
Biplot graph of the principal components analysis to relate seaweed composition and enzyme production during SSF. (AN: *A. niger*; AI: *A. ibericus*; AIR: acid insoluble residue; SFA: saturated fatty acids; MUFA: monounsaturated fatty acids; PUFA: polyunsaturated fatty acids; BGluc.: β-glucosidase).

The principal components analysis was carried out to observe the relationship between the seaweed composition and the production of enzymes during SSF (Figure 6). The correlating features were converted to the so-called principal components, which are themselves non-correlated. In the analysis, 41.7% of the variation of the results could be described by the PC1, while the PC2 explained 33.8% of the variation of the results. As can be observed, the red seaweeds were grouped in the same area of the graph due to their similar composition, that is, a high level of crude and soluble protein, phenols, arabinan, and C contents. On the other hand, the green seaweeds were not grouped in the same zone, which may be explained by differences in lipid content, since *U. rigida* had a low lipid level and *C. tomentosum* had a higher content. It is common to observe differences in the lipid content of green algae; Cardoso et al. [73] only observed similar contents among seaweeds of the *Ulva* species, however, other green seaweed species had different contents of lipids. Saigily et al. [74] also observed that the lipid content of *Codium* seaweed was twofold higher than that of the *Ulva* species. In addition, differences in salt and ash content were observed, which may be due to a higher washing effect in *U. rigida* than in *C. tomentosum*. The brown seaweed *A. esculenta* and green seaweed *C. tomentosum* were grouped together, correlating with the high lipid, ash, and carbohydrate contents, and some minerals (Cu, Na, Ca) and acid-insoluble residue (AIR). The PC2 positively correlated the enzyme production with the higher carbohydrate content, especially cellulose, rhamnan, free sugars, salts, and Mg, Mn, and Fe. The presence of polysaccharides can induce the production of carbohydrases by fungi under SSF [75]. The fungi produce these enzymes when cellulose and hemicelluloses are provided as carbon sources. The production of carbohydrases was correlated with the composition of *U. rigida*. This seaweed already showed the potential to produce carbohydrases, due to its composition [17]. On the other hand, the presence of minerals can stimulate enzymatic activities. Kumar et al. [76] observed that xylanase activity was stimulated by the presence of Mn and Fe. The positive effect of Mn on xylanase production was also observed in UV-mutated *A. niger* [77].

In addition, the PC2 negatively correlated the content of K, Zn, and Cu with the production of enzymes. The low effect of K on the xylanase activity was also detected in the xylanase produced by *Cladosporium oxysporum* [78]. The negative effect of the presence of Cu was also observed in the xylanase activity produced by *A. japonicus* [79].

## 4. Conclusions

This study shows the promising application of SSF for the biovalorization of seaweeds’ nutritional value and the production of high-value carbohydrases. The *A. ibericus* and *A. niger* successfully produced enzymes that modified the seaweeds’ structure, resulting in a protein increment, especially in the green seaweed *U. rigida*. Therefore, SSF is an environmentally friendly, cost-effective, and sustainable biovalorization approach that may result in nutritionally suitable seaweeds for aquafeeds. Future work must be carried out to study the viability of incorporating fermented seaweeds with less complex polysaccharide structures and enriched with carbohydrases in feeds for aquaculture fish species.

## Figures and Tables

**Table 1:** Physicochemical composition of the different seaweeds studied (% *w*/*w* dry seaweed).

Parameter	*Gracilaria* sp.	*P. dioica*	*C. tomentosum*	*U. rigida*	*A. esculenta*
Humidity	8.54 ± 0.36	9.08 ± 0.03	9.61 ± 0.29	15.5 ± 0.13	13.0 ± 0.79
Salt	18.1 ± 1.21	21.2 ± 0.85	31.4 ± 1.48	14.7 ± 0.74	15.3 ± 0.21
Ash	24.8 ± 0.00	19.2 ± 0.01	45.5 ± 0.05	23.2 ± 0.01	31.1 ± 0.02
Crude Protein	28.2 ± 0.00	23.9 ± 0.23	14.2 ± 0.04	14.6 ± 0.35	12.6 ± 0.16
Acid insoluble residue	9.71 ± 0.86	10.1 ± 0.01	16.2 ± 0.90	19.6 ± 0.77	19.0 ± 0.75
N	5.27 ± 0.27	4.51 ± 0.30	3.03 ± 0.07	3.21 ± 0.24	2.33 ± 0.12
C	30.9 ± 0.50	38.1 ± 0.44	21.7 ± 0.09	28.6 ± 0.29	28.5 ± 0.83
C/N	5.86	8.47	7.17	8.94	12.2
Carbohydrate	9.59 ± 0.08	12.9 ± 0.20	33.3 ± 1.27	29.8 ± 2.06	12.2 ± 1.35
Cellulose	3.98 ± 0.13	1.55 ± 0.44	10.6 ± 0.11	13.7 ± 1.45	7.08 ± 1.16
Agar	2.58 ± 0.01	4.84 ± 0.39	-	-	-
Arabinose	3.02 ± 0.02	6.49 ± 0.17	-	-	4.77 ± 0.88
Xylan	-	-	22.7 ± 2.38	3.47 ± 0.48	0.394 ± 0.18
Rhamnan	-	-	-	12.6 ± 1.59	-
Lipids	2.09 ± 0.16	1.92 ± 0.15	6.39 ± 1.12	1.91 ± 0.33	2.01 ± 0.17
Soluble proteins (g kg^−1^)	1.49 ± 0.11	4.06 ± 0.20	0.905 ± 0.11	1.45 ± 0.06	0.213 ± 0.01
Reducing sugars (g kg^−1^)	1.86 ± 0.11	2.44 ± 0.25	1.47 ± 0.21	4.96 ± 0.51	2.43 ± 0.00
Phenols (g GAE kg^−1^)	1.80 ± 0.02	4.47 ± 0.37	-	-	-

**Table 2:** Mineral composition of the different seaweeds (g kg^−1^ dry seaweed).

	Zn	Fe	Cu	Mn	Ca	Mg	K	Na	Se
*Gracilaria* sp.	-	2.87 ± 0.10	-	0.733 ± 0.03	2.32 ± 0.09	4.81 ± 0.15	93.5 ± 2.83	22.9 ± 0.56	-
*P. dioica*		0.394 ± 0.05		0.0768 ± 0.01	3.27 ± 0.02	5.61 ± 0.75	55.1 ± 1.02	24.0 ± 0.96	
*C. tomentosum*	0.112 ± 0.01	0.336 ± 0.04	0.978 ± 0.01	-	8.47 ± 0.54	15.7 ± 1.07	64.8 ± 7.57	103 ± 11.85	
*U. rigida*	-	3.58 ± 0.25	-	1.10 ± 0.15	6.31 ± 0.64	31.4 ± 2.70	30.4 ± 2.58	27.5 ± 2.79	
*A. esculenta*	1.51 ± 0.17	0.343 ± 0.03		-	15.8 ± 1.36	12.0 ± 1.20	103 ± 8.12	64.2 ± 5.94	

**Table 3:** Fatty acid profile of seaweeds (mg g^−1^ dry seaweed).

	*Gracilaria* sp.	*P. dioica*	*C. tomentosum*	*U. rigida*	*A. esculenta*
Caproic (C6:0)	0.307 ± 0.01	-	0.475 ± 0.02	-	0.308 ± 0.00
Caprylic (C8:0)	-	-	0.147 ± 0.00	-	0.119 ± 0.01
Capric (C10:0)	0.104 ± 0.01	0.0882 ± 0.00	0.101 ± 0.00	0.0870 ± 0.00	-
Lauric (C12:0)	0.0958 ± 0.00	0.0811 ± 0.00	0.544 ± 0.04	0.0849 ± 0.00	0.0899 ± 0.00
Myristic (C14:0)	0.432 ± 0.00	0.128 ± 0.00	1.13 ± 0.05	0.269 ± 0.00	1.08 ± 0.06
Palmitic (16:0)	2.77 ± 0.01	1.34 ± 0.07	14.3 ± 0.93	2.96 ± 0.20	2.77 ± 0.29
Palmitoleic (16:1 c6 n9)	0.113 ± 0.02	-	1.24 ± 0.06	0.193 ± 0.01	0.184 ± 0.01
Margaric (C17:0)	0.112 ± 0.00	0.105 ± 0.00	0.136 ± 0.00	0.112 ± 0.00	0.112 ± 0.00
Stearic (C18:0)	0.258 ± 0.01	0.222 ± 0.02	0.475 ± 0.02	0.319 ± 0.04	0.305 ± 0.09
Oleic (C18:1 c9 n9)	0.321 ± 0.02	0.288 ± 0.01	8.18 ± 0.55	0.927 ± 0.02	0.352 ± 0.02
Linoleic (C18:2 c9 c12 n6)	-	-	5.93 ± 0.34	-	-
GLA (C18:3 c6 c9 c12 n6)	-	-	1.25 ± 0.11	-	-
ALA (C18:3 c9 c12 c15 n3)	-	-	5.92 ± 0.35	0.216 ± 0.02	0.532 ± 0.03
ARA (C20:4 c5 c8 c11 c14 n6)	-	-	1.20 ± 0.11	-	-
EPA (C20:5 c5 c8 c11 c14 c17 n3)	-	-	1.10 ± 0.09	-	-
DHA (C22:6 c4 c7 c10 c13 c16 c19 n3)	1.18 ± 0.03	1.09 ± 0.00	5.66 ± 0.42	1.49 ± 0.03	1.36 ± 0.08
SFA	4.08 ± 0.01	1.96 ± 0.06	17.3 ± 1.06	3.83 ± 0.23	4.78 ± 0.43
MUFA	0.434 ± 0.00	0.288 ± 0.01	9.42 ± 0.61	1.12 ± 0.03	0.537 ± 0.03
PUFA	1.18 ± 0.03	1.09 ± 0.00	21.1 ± 1.42	1.71 ± 0.01	1.98 ± 0.05
Total fatty acids	5.69 ± 0.02	3.33 ± 0.07	47.8 ± 3.09	6.66 ± 0.22	7.21 ± 0.40

GLA: γ-linolenic acid; ALA: α-linolenic acid; ARA: arachidonic acid; EPA: eicosapentaenoic acid; DHA: docosahexaenoic acid; SFA: saturated fatty acids; MUFA: monounsaturated fatty acids; PUFA: polyunsaturated fatty acids.

**Table 4:** Enzyme production by solid-state fermentation of the different seaweeds (U g^−1^ dry fermented seaweed).

	Xylanase		Cellulase		β-glucosidase	
	*A. ibericus* MUM 03.49	*A. niger* CECT 2915	*A. ibericus* MUM 03.49	*A. niger* CECT 2915	*A. ibericus* MUM 03.49	*A. niger* CECT 2915
*G. gracilis*	3.32 ± 0.48 ^a^	1.94 ± 0.31 ^a^	22.1 ± 0.91 ^b^	124 ± 0.16 ^c^	7.17 ± 0.88 ^b^	7.86 ± 1.07 ^a^
*P. dioica*	3.40 ± 0.91 ^a^	8.94 ± 0.30 ^a^	62.1 ± 5.56 ^c^	81.6 ± 4.41 ^b^	6.17 ± 0.86 ^b^	7.64 ± 1.77 ^a^
*C. tomentosum*	0.729 ± 0.00 ^a^	-	2.25 ± 0.85 ^a^	-	0.197 ± 0.00 ^a^	-
*U. rigida*	61.5 ± 4.34 ^b^	327 ± 40.04 ^b^	19.8 ± 1.61 ^b^	101 ± 10.27 ^b^	27.5 ± 1.48 ^c^	20.4 ± 0.17 ^b^
*A. esculenta*	-	3.53 ± 1.23 ^a^	4.59 ± 0.24 ^a^	1.78 ± 0.36 ^a^	3.75 ± 0.36 ^b^	4.88 ± 0.30 ^a^

Results are presented as the mean (n = 3) ± SD. Different letters in the same column indicate significant differences between seaweed species (*p* < 0.05).

## Data Availability

The data are available from the corresponding author.
